# Long RNA-Mediated Chromatin Regulation in Fission Yeast and Mammals

**DOI:** 10.3390/ijms23020968

**Published:** 2022-01-16

**Authors:** Matthew W. Faber, Tommy V. Vo

**Affiliations:** Department of Biochemistry and Molecular Biology, College of Human Medicine, Michigan State University, East Lansing, MI 48824, USA; faberma1@msu.edu

**Keywords:** long noncoding RNA, long regulatory RNA, chromatin, gene neighborhood, heterochromatin, higher-order chromosomal architecture, YTH-family protein, *S. pombe*, mouse, human

## Abstract

As part of a complex network of genome control, long regulatory RNAs exert significant influences on chromatin dynamics. Understanding how this occurs could illuminate new avenues for disease treatment and lead to new hypotheses that would advance gene regulatory research. Recent studies using the model fission yeast *Schizosaccharomyces pombe* (*S. pombe*) and powerful parallel sequencing technologies have provided many insights in this area. This review will give an overview of key findings in *S. pombe* that relate long RNAs to multiple levels of chromatin regulation: histone modifications, gene neighborhood regulation in *cis* and higher-order chromosomal ordering. Moreover, we discuss parallels recently found in mammals to help bridge the knowledge gap between the study systems.

## 1. Introduction 

From yeast to mammals, eukaryotic genomes can be pervasively transcribed depending on developmental or environmental states. It is estimated that most of the *Schizosaccharomyces pombe* (fission yeast, *S. pombe*) and human genomes are transcription-competent [1,2]. Although protein-encoding genes make up a tiny minority of all transcribed genomic units, they have historically garnered the most research attention. However, in light of recent advances in next-generation sequencing and genome editing approaches, there has been increasing engagement in illuminating the functional relevance of genes encoding regulatory RNAs. These include noncoding RNAs and bifunctional RNAs with dual coding and noncoding attributes.

The transcriptional products of noncoding genes can be broadly classified as small noncoding or long noncoding RNAs (lncRNAs). Small noncoding RNAs are less than 200 nucleotides in length and are comprised of major subclasses, including microRNAs (miRNAs), short interfering RNAs (siRNAs), tRNA-derived small RNAs (tsRNAs) and piwi-interacting RNAs (piRNAs). Their roles in transcriptome and chromatin regulation have been extensively reviewed elsewhere and will not be the focus of this review [3,4,5,6,7,8]. Long RNAs (>200 nucleotides in length) called long noncoding RNAs (lncRNAs) are not believed to be translated into protein. Compared to messenger RNAs (mRNAs), many lncRNAs are poorly conserved in sequence, are less stable and primarily reside within the nucleus. Within yeast, plants and animals, the number of genes encoding lncRNAs vastly outweighs the number of mRNA-encoding genes [9,10,11,12], suggesting that either there is substantial non-functional transcriptional noise across eukaryotes or that there remain many functional RNAs still awaiting characterization.

Nevertheless, it has been debated that some annotated lncRNAs might be misannotated and can be translated [13,14,15]. This idea was supported by ribosome-profiling work showing that many human and mouse lncRNAs can interact with cytoplasmic ribosomes [16,17]. However, it was unclear if the interactions promote the synthesis of functional peptides, RNA decay, or other processes. It is certainly possible that some long RNAs may be bifunctional with coding and noncoding functions [18,19,20]. For example, the first discovered mammalian bifunctional RNA (bifRNA) called Steroid Receptor RNA Activator (SRA) regulates gene expression as an RNA [21] and nuclear receptor complexes as a protein [22]. This review will focus on chromatin regulatory roles of long RNAs, irrespective of whether they might be *bona fide* lncRNAs or bifRNAs.

In recent years, the combination of facile genetics, molecular biology and biochemistry with high-throughput sequencing approaches has enabled the fission yeast *S. pombe* to become an outstanding model for understanding the regulatory functions of nuclear long RNAs. Excitingly, studies in mammalian mouse and human models are also beginning to reveal similar mechanisms of long RNA action at the chromatin level. Here, we review various chromatin-level modes of long RNA-mediated gene regulation in *S. pombe* and mammals, with special attention to their similarities. In particular, we will focus on the mechanisms by which long RNAs orchestrate control over epigenetic modifications, transcription termination and higher-order chromatin architectures. Altogether, this review will summarize our latest knowledge on how fission yeast and mammals leverage long RNAs to elicit conserved mechanisms of genome control.

## 2. Tools to Identify Long Regulatory RNAs

Next-generation sequencing (NGS; Illumina, Roche 454) and third-generation sequencing (TGS; PacBio, Nanopore) capabilities have dramatically enabled us to discover long regulatory RNA species. Using strand-specific RNA next-generation sequencing (NGS) to massively produce short sequencing reads is currently the most prominent approach to empirically detect entire RNA repertoires within cells. The short reads are then bioinformatically mapped onto reference transcriptomes, using various open-sourced short-read mapping software [23] to infer any expressed RNA. The mapped RNAs are usually post-characterized bioinformatically or experimentally using RNA probing assays such as Northern blotting. The major advantage of this short-reads sequencing approach is that genomic regions which produce any RNA(s) can be detected with high sensitivity and relatively low background, particularly compared to lower resolution approaches such as tiling arrays. However, it is often challenging to differentiate long RNAs from shorter overlapping ones, which poses a significant problem for small-genome organisms, such as fission yeast, with many overlapping expressed genes. More recently, the emergence of long-read third generation sequencing (TGS), such as PacBio SMRT and Oxford Nanopore, appears to remedy the identification problems faced by short-read sequencing methods [24,25]. It is now possible to sequence entire RNAs from 5′ to 3′ ends and directly characterize all present RNA species. The main limitations of TGS methods include the higher error rates, costs and few pre-packaged bioinformatic analysis tools when compared to NGS methods [24].

Post-identification, a range of tools are available to assess the regulatory potential of lncRNA transcripts. Aside from a range of computational tools to predict regulatory functions of lncRNAs [26,27,28], there are various experimental approaches to more directly test for regulatory potential. Genetically, lncRNAs can be ablated or knocked down to assess the impact on certain phenotypes like gene expressions. These methods include using CRISPR-Cas9, CRISPR-Cas13, or antisense oligonucleotides [29,30,31]. Genome-wide approaches can also be used to propose regulatory functions via indirect means. Due to the shear abundance of available approaches, Table 1 represents an inexhaustive list of these methods that measure different biophysical properties of lncRNAs including RNA–DNA interactions [32,33,34,35,36], inter-RNA–RNA interactions [37,38,39,40], RNA–protein interactions [41,42,43,44,45] and RNA localization [46,47,48]. More comprehensive reviews elsewhere have been performed on methods that map complex RNA-chromatin interactions [49,50]. Nevertheless, direct genetic and biochemical manipulation of lncRNAs remains the most direct route to elucidating their functions [51].

Long bifRNAs with dual coding and noncoding functions are more complicated to work with because they require dissecting functions of the RNA from the protein that they encode [52]. Genetic inspection of these transcripts often requires introducing synonymous mutations that would ideally preserve coding potential while affecting noncoding functions. However, doing so may still affect translation rates, and thus protein levels, and confuscate biological interpretations [53]. Alternatively, the transcripts can be characterized using the genome-wide approaches described above to interrogate lncRNA functions. Understanding these long RNAs from multiple molecular perspectives are required to determine their regulatory potential.

## 3. Long Regulatory RNAs and Their Protein Partners

It is often that long RNAs, such as lncRNAs and others, may function by regulating transcript-bound protein partners’ activities [51]. This can be especially appreciated in the fission yeast *S. pombe* through many studies on the Mmi1 RNA-binding protein, as will be our focus below. We will also highlight striking similarities that have only recently been realized between the regulatory functions of Mmi1-targeted transcripts and RNA-targets of YTH Domain Containing 1 (YTHDC1), which is the mammalian homolog of yeast Mmi1.

Mmi1 belongs to the family of conserved YT521-B homology (YTH) domain proteins. Its YTH protein domain, located at the C-terminus of *S. pombe* Mmi1, is essential for directly binding to RNA transcripts [54,55,56]. Initially, Mmi1 was discovered by Yamamoto and colleagues to genetically interact with meiotic genes to trigger timely RNA degradation and regulate the mitosis–meiosis transition [57]. Mmi1 directly binds to long transcripts from these loci by recognizing an RNA motif called the Determinant of Selective Removal (DSR). The DSR region is characterized by the consensus hexamer motif U(U/C)AAAC [58]. More recent studies using DNA microarray and NGS approaches discovered that Mmi1 can associate with many other DSR motif-containing long RNAs, including the lncRNAs *meiRNA* [59], *nam* [60] and *mamRNA* [61]. The ability for Mmi1 to interact with RNAs positively correlates with how many closely-spaced repeats of the DSR motif there are within a single transcript [58]. While the Mmi1-RNA associations were initially thought of as unidirectional [57], whereby Mmi1 binding simply leads to transcript elimination, more recent work has shown that the interactions play critical roles in targeted chromatin regulation and nearby-gene control, as discussed in the sections below.

A combination of genetic analyses and protein-centric studies has revealed that transcripts bound by Mmi1 are associated with many additional protein factors. These include the Clr4 methyltransferase that is responsible for the post-translational modification of chromatin with di-methylation of histone H3 lysine-9 (H3K9me2), RNA 3′-end processing and transcription termination (Pab2, Cleavage and Polyadenylation Factor [CPF], Dhp1), RNA 5′ binding and splicing (Pir2, Cwf10) and RNA degradation (Erh1, Mtl1-Red1 core (MTREC), Ccr4-NOT complex, Rrp6) [60,62,63,64,65,66,67,68,69,70,71]. A possible function of these RNAs is to guide Mmi1 with various combinations of other factors to specific genomic loci and to influence gene expression. 

In mammals, the Mmi1-homolog YTHDC1 directly binds to the *N*^6^-methyladenosine (m^6^A) post-translational modification of long RNAs [72]. Some of its well-known RNA substrates have known roles in chromatin regulation and protein complex assemblies. These long RNA targets include the lncRNAs metastasis associated lung adenocarcinoma transcript 1 (*MALAT1)*, HOX transcript antisense RNA (*HOTAIR)* and X-inactive specific transcript (*XIST)*. *MALAT1* associates with actively transcribed chromatin regions and positions them near nuclear speckles [73,74]. These nuclear bodies are gene expression regulatory factories that contain clusters of RNAs, chromatin remodelers, RNA processing factors and more [75]. A recent study by Wang et al. demonstrated that m^6^A-modified *MALAT1* and YTHDC1 are needed for the chromatin-speckles association and gene activation [74]. In contrast, the lncRNA *HOTAIR* interacts with YTHDC1, including at the m^6^A modification of *HOTAIR* residue A783 [76] to localize to chromatin and mediate gene repression. The third mammalian lncRNA *XIST* has many m^6^A-modifiable sites, with at least 78 residues identified by m^6^A-mapping using m^6^A iCLIP [77], and interacts with factors such as YTHDC1, SAF-A, SPEN and PRC2 at the inactive X-chromosome [77,78,79,80]. There, they promote the assembly of tri-methylated lysine-27 of histone H3 (H3K27me3) and transcriptional repression. Interestingly, recent work has shown that *XIST* RNA can promote the compartmentalization of specific ribonucleoprotein complexes at chromatin to mediate its silencing functions [81]. Although the exact modes of RNA recognition and binding are different between the homologs *S. pombe* Mmi1 and mammalian YTHDC1, which bind to DSR motifs and m^6^A, respectively [54,55], these conserved proteins can use long RNAs to mediate similar repressive functions. This suggests that the partnership between long RNAs and YTH-domain proteins to regulate chromatin landscapes may be evolutionarily conserved between fission yeast and mammals. 

## 4. RNA-Mediated Chromatin Modifications

Histones within chromatin are subject to post-translational modifications, including methylation, acetylation, ubiquitination, citrullination and phosphorylation. These modifications can act as molecular platforms to recruit gene regulatory factors, such as histone H3 lysine-9 tri-methylation (H3K9me3) recruiting Heterochromatin Protein 1 (HP1) family proteins [82,83,84,85,86]. Additionally, modifications can be epigenetic markers that regulate gene expression in manners that are heritable [3,86,87,88].

In *S. pombe*, small and long regulatory RNAs have been linked to targeted histone methylations, particularly H3K9me2 and H3K9me3. In this yeast and across higher eukaryotes, these modifications are the hallmarks of chromatin domains called heterochromatin [3,86,87,89,90]. Heterochromatin regions can have condensed physical structures [91] that serve to be inhibitory to *trans*-acting factors. The two broad types of heterochromatin that exist are called constitutive and facultative. In *S. pombe*, constitutive heterochromatin is marked by the presence of H3K9me2/3 and is predominately nucleated by small interfering RNAs (siRNAs) that derive from repetitive transcripts [3,92]. Conversely, facultative heterochromatin is preferentially enriched for H3K9me2 that depends on DNA-binding [93] and RNA-binding factors [62,63,64,65,66,70,94,95,96]. 

Recent works in fission yeast have provided mechanistic insights into how long RNAs promote H3K9me2 modifications at constitutive and facultative heterochromatin. At pericentromeric constitutive heterochromatin, the existence of distinct regulatory lncRNAs called noncoding RNA associated with Mmi1 (*nam*) was identified [60]. These transcripts associate with Mmi1 through their DSR motifs. In particular, *nam* transcripts called *nam5/6/7* were found to be produced from pericentromeric *dh* repeats. Mmi1, the nuclear exosome subunit Rrp6 and the CPF subunit Swd22 target these RNAs to promote H3K9me2 at pericentromeres [60,66,70]. It was proposed that transcription termination of these lncRNAs helps to promote H3K9me2 [60], a model supported by prior observations that an essential transcription termination factor, Dhp1, is involved in promoting pericentromeric H3K9me2 [64,96]. Subsequently, it was recently shown that the CPF core subunit Iss1, which Mmi1 can recruit, localizes to the 3′ ends of pericentromeric *nam5/6/7* genes [66]. Both CPF and Dhp1 gene-targeting in *S. pombe* is often needed to mediate transcription termination [97]. To demonstrate the importance of termination on constitutive heterochromatin, it was shown that double mutations in the RNA interference (RNAi) pathway with mutation of either Mmi1 or CPF led to cumulative increases in *nam5/6/7* RNA [60] or total ablation of pericentromeric H3K9me2 [66,70], respectively. However, the RNAi pathway appears to play a more prominent role in pericentromeric H3K9me2 since mutation of RNAi factors diminish H3K9me2 levels more than mutations in only the Mmi1 pathway [60,66]. The Mmi1 pathway might serve as a backup mechanism to ensure the presence of heterochromatic features at the pericentromeres when RNAi is inactive. This might be important to prevent detrimental centromeric instability due to enhanced recombination in the absence of protective heterochromatin [98]. Hence, in addition to small RNAs, lncRNAs also play an important role in promoting the deposition of repressive histone modifications at constitutive heterochromatin in the fission yeast.

**Figure 1:**
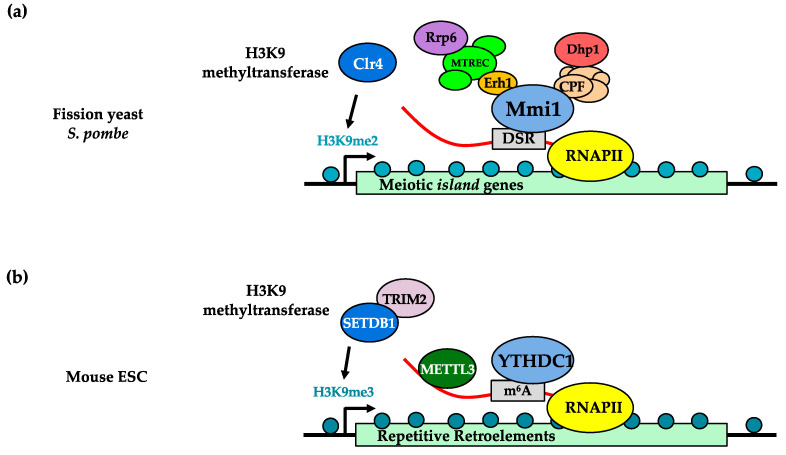
Histone H3 lysine 9 (H3K9) methylation promoted by YTH-family proteins. (**a**) In *S. pombe*, Mmi1 binds Determinant of Selective Removal (DSR) sequence motifs of meiotic RNAs derived from islands and engages with additional RNA processing and chromatin modification factors to trigger deposition of H3K9me2. (**b**) In mouse embryonic stem cells, YTHDC1 binds to certain m^6^A-modified RNAs to promote chromatin-association of m^6^A-writer METTL3. This promotes SETDB/TRIM28-mediated deposition of repressive H3K9me3 modifications.

In addition to constitutive heterochromatin, Mmi1-targeted RNAs in *S. pombe* have been shown to be important for promoting H3K9me2 at facultative heterochromatin domains called *islands* [62]. The levels of H3K9me2 modifications at these regions vary depending on environmental or developmental conditions and promote the repression of nearby genes [62,93]. Furthermore, silencing of the euchromatic *ura4* gene can be enforced by the artificial insertion of the gene into a locus near an endogenous facultative *island* [66,93,99]. This is a hallmark Position Effect Variegation (PEV) phenotype characteristic of heterochromatin [100]. In particular, at a subset of *islands* that comprise meiotic genes, transcription during mitotic growth, which is paradoxically when these genes should be repressed, is required to promote H3K9me2 chromatin modifications. The transcription requirement for H3K9me2 at these *islands* was previously demonstrated by the insertion of a premature transcription termination sequence within the promoter of the *ssm4* meiotic gene that resulted in the loss of H3K9me2 at the *ssm4 island* locus [62]. Transcription of these meiotic genes produces DSR-containing long RNAs that cluster within the nucleus [94,101] and recruit the factors Mmi1, Erh1, MTREC, CPF, Dhp1, Rrp6 and the H3K9 methyltransferase Clr4 (Figure 1a) [63,64,66,70,94]. These factors have been implicated in various nuclear processes, including RNA degradation, RNA export, RNA splicing, transcription termination and histone methylation. Recent work has begun to dissect the precise molecular interactions between these distinct factors that ultimately promote the assembly of facultative H3K9me2 in *S. pombe*. The C-terminal YTH domain of Mmi1 can make direct contacts with the DSR motifs of these meiotic long RNAs [54,55]. On the N-terminus of Mmi1 is a domain that makes contact with the highly conserved Erh1 nuclear protein [94] to form the Erh1-Mmi1 complex (EMC) [65]. In vitro, EMC stably exists as a tetrameric complex comprising a dimer of Mmi1-Erh1 dimer (two Mmi1 monomers, two Erh1 monomers). This complex likely binds to DSR-containing meiotic RNAs and modulates the recruitment of other protein factors to prevent untimely meiotic gene expressions [94,101,102]. MTREC appears to be an intermediary complex that physically links Mmi1 to Rrp6 and Clr4 [63,103]. This linkage is crucial for promoting H3K9me2 at *islands* and for the degradation of the meiotic transcripts [62,68,103]. In addition, factors involved in transcription termination and RNA 3′-end processing were shown to be essential for *island* H3K9me2 since genetic mutation of those factors, such as in Dhp1, Swd22 and Ssu72, abolished those chromatin modifications [64,66,96]. Recruitment of those factors to the meiotic *islands* requires Mmi1, suggesting that they may act downstream from or concurrently with Mmi1-RNA binding [66]. Altogether, these *island*-derived long RNAs promote local chromatin modification by facilitating the recruitment of protein factors that ultimately regulate the chromatin loci from which these RNAs originate.

Recently, work from various groups using mouse embryonic stem cells has shown evidence suggesting that YTHDC1, the mammalian homolog of fission yeast Mmi1, might similarly play important roles in promoting repressive chromatin modifications through long RNA recognition. It was discovered that YTHDC1 binds to m^6^A-marked retrotransposons to promote assembly of SETDB1-mediated H3K9me3 and repression of embryonic reprogramming genes, including *DUX* and *MERV* [104]. Separately, Xu et al. found that YTHDC1 binds to chromatin-associated endogenous retroviral (ERV) RNAs that are m^6^A-modified [105]. In these cases, YTHDC1-RNA binding stabilizes the chromatin association of the m^6^A-writer METTL3, which then recruits the H3K9me3-promoting TRIM28-SETDB1 complex to modify the underlying chromatin domains (Figure 1b). In addition to promoting repressive H3K9me3 modifications, a third group showed that YTHDC1 mediates recruitment of Polycomb Complex 2 (PRC2) to chromatin loci [106]. This suggests that YTHDC1 may also help trigger the assembly of repressive H3K27me3, which was recently observed at a reporter gene that expressed m^6^A-modified long RNA [107]. Finally, Quinodoz et al. found that minor and major satellite-derived RNAs cluster at HP1/H3K9me3-rich pericentromeric regions [32]. These RNAs were necessary to recruit HP1 clusters, suggesting that they may act similar to fission yeast *nam* lncRNAs to promote pericentromeric heterochromatin. In contrast to these findings, another recent study also indicated that YTHDC1 could promote H3K9me2 demethylation to help activate gene expression [107]. It is possible that YTHDC1 is multi-functional and assumes different functional identities depending on the target loci. Altogether, recent and emerging studies are beginning to show that mammalian YTHDC1 and fission yeast Mmi1 are important long RNA interactors that facilitate chromatin modifications for proper gene control. These chromatin-level functions add additional complexity to YTH family proteins, which have historically been better characterized as RNA processing factors.

## 5. Regulation of Nearby Gene Transcription by Long RNAs

Virtually all organisms need to address how to coordinate the expression of closely-spaced and sometimes overlapping genes [108]. In these cases, cells must be able to parse distinct genes that may share regulatory features, such as stretches of DNA that could be a promoter of one gene and an open reading frame of another or be influenced by a common transcriptional event. This is a problem that is faced in the human genome, where approximately 25.8% of coding genes overlap with adjacent genes [109], of which approximately 52.4% of overlapping pairs consist of tandem genes that are transcribed in the same direction and could potentially experience transcriptional interference [110]. In more gene-dense organisms such as *Saccharomyces cerevisiae* and *S. pombe*, where the median intergenic distances are ~366 bp and ~441 bp, respectively [111], lncRNAs represent one approach to regulate neighboring genes. For instance, in *S. cerevisiae*, lncRNAs called upstream-initiating transcripts (UPS) have recently been suggested to regulate transcription of nearby downstream rDNA genes [112]. In *S. pombe*, three example lncRNAs that have been studied in detail are *nam1*, *rse1* and *prt* [60,63,113], which regulate the nearby genes *byr2*, *ste11* and *pho1*, respectively. Studies on the functions of *nam1* and *rse1* lncRNAs were recently reviewed by Andric and Rougemaille [114] and will not be discussed. Below, we review the current understanding of how the *prt* lncRNA represses the downstream *pho1* coding gene.

Pho1 is a phosphate-responsive secreted acid phosphatase that is involved in phosphate uptake. Its gene expression requires the transcription factor Pho7, which directly binds to the upstream *pho*1 promoter [115,116,117,118]. *Pho1* is considered a model nutrient-sensing gene because its expression is inversely correlated with phosphate abundance in the cellular environment [63,69,119]. A key question in the field has been how is *pho1* expression controlled in response to available phosphate? From the perspective of cellular phosphates, phosphorylation of RNA polymerase II C-terminal domain (CTD) and phosphate-rich inositol pyrophosphates have been shown to affect *pho1* expression [119,120,121,122,123]. Interestingly, these factors have been shown to affect a nearby *prt* lncRNA gene, which is situated immediately upstream of the *pho1* coding gene and is transcribed in the same direction as *pho1*. The *prt* promoter is within 110 bp upstream of the *prt* transcribed start site [124]. The *prt* transcription termination region is located proximal to the promoter of the *pho1* gene [66,69,116]. While it is well-documented that the *prt* noncoding gene negatively regulates *pho1* expression [63,69,124,125], recent studies have focused on the precise mechanisms by which the *prt* lncRNA gene represses expression of the downstream *pho1* gene. 

Evidence indicates that *prt* gene transcription promotes *pho1* repression. Ablation of the *prt* promoter or the *prt* transcribed region completely prevents *prt* transcription and elevates *pho1* expression [63,69,124]. Similarly, Yague-Sanz et al. had recently reported that slowing down transcription kinetics by a point mutation within RNA polymerase II (RNAPII) can affect *prt* transcription and increase *pho1* expression [126]. Moreover, extensive studies from Shuman and colleagues have shown that the phosphorylation status of RNAPII impacts the expression of *pho1* [120,127,128]. For example, phosphorylation of Ser7 and Ser5 of RNAPII carboxy-terminal domain (CTD) was shown to be required for the repression of *pho1* [125]. Subsequently, it was suggested that preventing Ser7 phosphorylation derepresses *pho1* by promoting early CPF-mediated transcription termination of the upstream *prt* gene [127]. Work from other groups has also shown that direct transcription termination of *prt* by CPF and Dhp1 [66,69] is likely crucial to enable the Pho7 transcription factor to activate transcription of the downstream *pho1* gene [116,117]. Altogether, transcription-related factors at *prt* appear important for the nearby regulation of *pho1* gene expression in *cis*.

**Figure 2:**
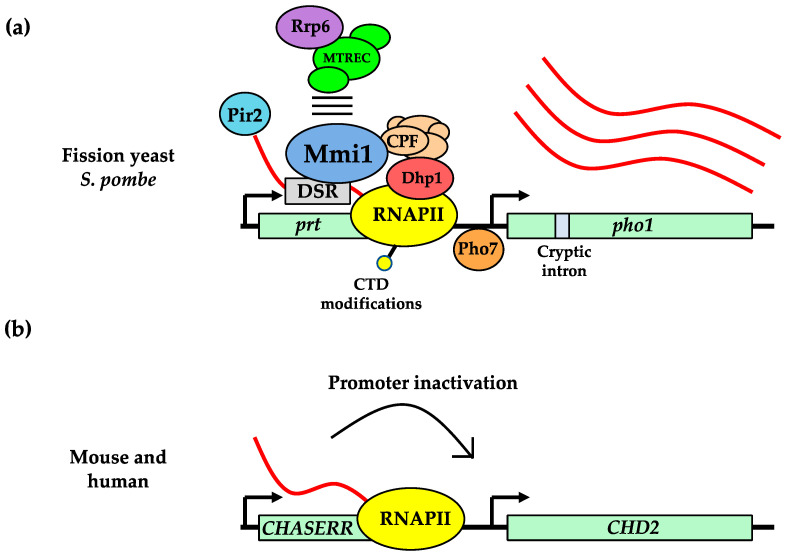
Gene expression control in *cis* by nearby lncRNAs. (**a**) In *S. pombe*, Mmi1 and additional protein factors, that are implicated in RNA processing and transcription regulation, associate with *prt* gene locus and lncRNA to affect expression of the downstream *pho1* gene locus. (**b**) In mouse and human cells, the lncRNA *CHASERR* transcriptionally represses the downstream *CHD2* gene through mechanisms that are currently unclear.

Increasing evidence suggests that the *prt* lncRNA has a direct function in regulating its own expression and that of the nearby *pho1* gene. The *prt* transcript has DSR motifs and is bound by Mmi1 protein. Deleting either the DSR motifs or the *mmi1* gene leads to *prt* transcription that continues unabated throughout the *pho1* gene body [63,65,66,69,94]. Mmi1 directly acts on *prt* transcript to control downstream *pho1* expression because studies using electrophoretic mobility shift assays have confirmed that Mmi1 can only bind *prt* RNA and not *prt* ssDNA or dsDNA [124]. These findings, along with the fact that Mmi1 associates with the *prt* gene locus in chromatin immunoprecipitation assays [65], suggest that the *prt* lncRNA directly recruits Mmi1 to the vicinity of the underlying chromatin locus. There, Mmi1 recruits CPF to promote early transcription termination of *prt* to prevent transcriptional readthrough into the *pho1* locus, thereby allowing proper *pho1* mRNA expression [66,121]. Given that RNAPII CTD phosphorylation statuses also affect *prt* termination [120,127,128], it is conceivable that the *prt* lncRNA and the transcriptional process of making the lncRNA both make distinct contributions for modulating the nearby *pho1* gene (Figure 2a).

In addition to the Mmi1-binding DSR motifs, deep RNA sequencing recently revealed that the *prt* long RNA can also contain a cryptic intron [67]. The intron helps link the splicing factor Cwf10 with the 5′ cap-binding protein Pir2 to promote *pho1* repression. This newfound protein network also includes RNAi, the Clr3 histone deacetylase and facilitates chromatin transcription (FACT) complex. Hence, the *prt* lncRNA contains multiple regulatory features. It remains to be seen precisely how *prt* DSR and cryptic intron signals are coordinated to measure *pho1* expression.

In mouse and human, a few lncRNAs (e.g., *XIST*) have been characterized to regulate the transcription of gene neighborhoods [129]. However, the recently characterized *CHD2* adjacent suppressive regulatory RNA (*CHASERR*) lncRNA appears most similar to yeast *prt*. It is one of the most conserved lncRNAs across vertebrates and is situated in tandem upstream of the nearby *CHD2* gene, which encodes for a DNA helicase [130,131]. Similar to the *prt*-*pho1* relationship in fission yeast, Rom et al. found that disruption of *CHASERR* led to derepression of the downstream *CHD2* gene [131,132]. *CHASERR*-mediated repression of *CHD2* likely occurs in *cis* because exogenous expression of *CHASERR* in *trans* was unable to repress *CHD*2 expression. Still, artificial activation of the lncRNA at its native chromosomal location could repress *CHD2*. Interestingly, targeting *CHASERR* using antisense oligonucleotides also led to elevated *CDH2* expression, suggesting that *CHASERR* lncRNA itself can repress its nearby gene (Figure 2b). Another study predicted that *CHASERR* might be able to co-transcriptionally base-pair with multiple different nascent RNAs, potentially regulating non-*CHD2* genes in *trans* [133]. Altogether, it is becoming clear that long RNAs can have important roles in regulating the transcription of neighboring genes in yeast and mammals.

## 6. Higher-Order Chromosomal Structuring by lncRNAs

Spatiotemporal chromatin regulation is emerging as a topic of great interest due, in part, to the expanding capabilities to determine chromatin architectures and localizations with high resolution [134,135]. Recent studies in fission yeast and mammals have suggested that long noncoding RNAs and their protein partners have active roles in this process. A clear example in *S. pombe* is the *meiRNA* lncRNA that is transcribed from the *sme2* gene [136]. In a similar vein, recent work on so-called architectural RNAs, such as *XIST* and *FIRRE*, in mouse or human cell lines provide evidence that mammalian lncRNAs may also play essential roles in higher-order chromosomal structuring [137,138,139,140,141]. The precise roles of these RNAs on higher-order chromatin topology have been a topic of recent interest.

**Figure 3:**
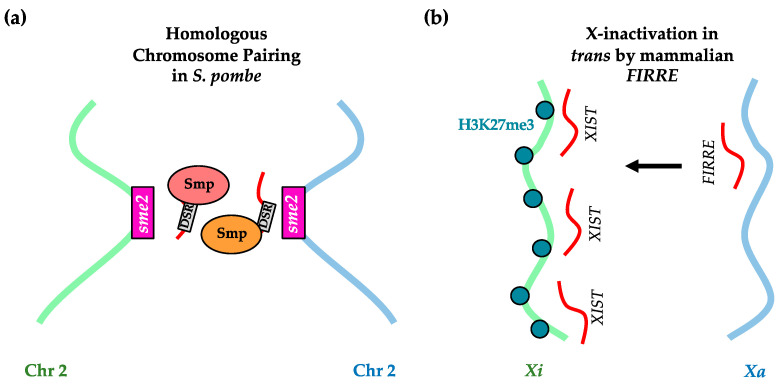
Intra-chromosomal regulation by long RNAs. (**a**) In *S. pombe*, DSR motif-containing RNAs from homologous chromosomes recruit *sme2*-assocating proteins (SMPs) to form nuclear clusters that help to bring the homologous chromosomes together. (**b**) In mammalian cells, the architectural lncRNA *FIRRE* that is expressed from the active X chromosome (*Xa*) promotes H3K27me3 and *trans* inactivation of the X chromosome (*Xi*).

Work from Ding et al. using *S. pombe* has revealed a dependence on lncRNA-centered ribonucleoprotein complexes for one of the most striking examples of chromosomal restructuring: homologous chromosome pairing during meiosis [136,142]. This process begins after two haploid cells fuse their nuclei to reconstitute a diploid cell with two copies of every chromosome. The authors initially noticed from live-cell microscopy that a protein called Mei2, which indirectly associates with the *sme2* gene locus through binding the *sme2*-derived *meiRNA* [143], only appears as a single focus in diploid cells despite having two homologous alleles of *sme2* [136,144]. Subsequently, they found that the Mei2 focus in diploid meiotic cells was a marker for paired homologous *sme2* alleles but was not a requirement because disruption of the Mei2-*meiRNA* interaction did not impede homologous pairing of the *sme2* alleles. A recent follow-up study identified additional protein factors whose localization mirrored Mei2. Some, such as Seb1, Pcf11 and Rhn1, were important for *sme2* homologous allele pairing [142]. These factors were generically referred to as *sme2* RNA-associated protein (Smp) and were enriched for known functional roles in RNA polyadenylation or transcription termination (Figure 3a). In addition, the authors found that another chromatin-associated lncRNA called *omt3* is required for the proper homologous pairing of chromosome I. The pairing was mediated by the lncRNAs, specific Smps and possibly liquid–liquid phase compartments [142]. These findings suggest that additional chromatin-associated ribonucleoprotein complexes might exist to target other chromosomal regions for proper chromosomal pairing/restructuring during meiosis.

In mammals, the inactivated X chromosome (*Xi*) is a prime model for understanding how lncRNAs similarly regulate chromatin. Within female cells that carry two X chromosomes, one is stably inactivated so that X-linked gene dosage is equivalent to XY male cells, where the sole X chromosome is active [145,146]. The *XIST* lncRNA that is transcribed from *Xi* is critical for inactivation by first nucleating repression at the *XIST* locus and then spreading along the *Xi* to expand the repression zone [80,81,147]. This expansion mechanism is dependent on various repeat motifs present within *XIST*, as reviewed elsewhere [92]. *XIST* recruits PRC2 to deposit repressive H3K27me3 marks throughout *Xi* [148,149]. *XIST* lncRNA also repels binding of chromosomal remodeling proteins, such as cohesin, to enforce *Xi*-specific chromosomal structures [138]. In addition to *XIST*, a more recent study from Fang et al. identified the *FIRRE* lncRNA, transcribed from the active X chromosome (*Xa*), as a long-range regulator of the homologous *Xi* [139]. While it minimally affects *XIST*-coating of the *Xi*, *FIRRE* does promote repressive H3K27me3 and localization of the *Xi* near the nuclear periphery or the nucleolus (Figure 3b). Furthermore, it promotes the binding of another architectural protein called CCCTC-binding factor (CTCF) to the *Xi*, with possible implications on *Xi* chromosomal structure or localization [139,150]. More globally, *FIRRE* has also been suggested to promote H3K27me3 at autosomal chromatin regions [137]. Given the known functional links between heterochromatic maintenance, chromosomal structures and chromosomal localization at the nuclear periphery [84,141,151,152], it will be interesting to further determine the exact roles of lncRNAs, such as *FIRRE*, within the context of these relationships. 

## 7. Conclusions

The fission yeast *S. pombe* has been an important model for understanding how long RNAs shape chromatin landscapes, from the individual locus level to much broader chromosomal regions. The striking conservation of these RNA roles in fission yeast and mammals, representing ~1 billion years of evolution, strongly suggests that long RNAs and their interacting protein partners are crucial mediators between nuclear chromatin and the cellular environment. With major advances in sequencing technologies, the identities and functions of transcriptomes are beginning to emerge in unexpected ways. For example, we now understand that *nam* lncRNAs in yeast are functional transcripts with significant implications for gene repression. The recent studies highlighted here invite numerous additional questions that could be addressed with currently available technologies. What are the sequenced-based and non-sequenced-based features of long RNAs that confer functional specificity? When do cells employ specific RNA-mediated regulatory mechanisms? With cells often expressing multiple copies of an RNA, how are those copies distributed across the genome for regulatory or non-regulatory purposes?

With the introduction of whole-genome sequencing over two decades ago, it was believed that all genome biology would be solved within a matter of years. Today, that sentiment could not be further from the truth. Very likely, higher quality, higher depth sequencing within the context of different genomic, developmental or environmental conditions will illuminate new strategies by which nature regulates their genomes. This will undoubtedly expand our knowledge on the sophisticated mechanisms by which transcriptional RNA products loop back to regulate the underlying DNA templates.

## Figures and Tables

**Table 1:** Summary of representative methods to detect long noncoding RNA (lncRNA) interactions or localization.

Method Full Name	Method Abbrev.	Measurement Type	References
RNA and DNA Split-Pool Recognition of Interactions by Tag Extension	RD-SPRITE	RNA–DNA interaction	[32]
Chromatin-Associated RNA sequencing	ChAR-seq	RNA–DNA interaction	[33]
RNA–DNA proximity ligation technique	Red-C	RNA–DNA interaction	[34]
Chromatin Isolation by RNA Purification sequencing	ChIRP-seq	RNA–DNA interaction	[35]
RNA Antisense Purification	RAP	RNA–DNA interaction	[36]
Ligation of interacting RNA followed by high-throughput sequencing	LIGR-seq	RNA–RNA interaction	[37]
Cross-linking Ligation and Sequencing of Hybrids	CLASH	RNA–RNA interaction	[38]
RNA In situ Conformation sequencing	RIC-seq	RNA–RNA interaction	[39]
Cross-linking Of Matches RNA And Deep Sequencing	COMRADES	RNA–RNA interaction	[40]
RNA Tagging	-	RNA–protein interaction	[41]
RNA Immunoprecipitation Sequencing	RIP-seq	RNA–protein interaction	[43]
Yeast three-hybrid	Y3H	RNA–protein interaction	[45]
Photoactivatable-Ribonucleoside-Enhanced Crosslinking and Immunoprecipitation	PAR-CLIP	RNA–protein interaction	[44]
Proximity Labeling	-	RNA–protein interaction	[42]
APEX2-mediated Proximity biotinylation of Endogenous RNAs	APEX-seq	RNA localization	[46]
Turbo Fluorescence In Situ Hybridization	Turbo FISH	RNA localization	[47]
Sequential Fluorescence In Situ Hybridization	SeqFISH	RNA localization	[48]

## Data Availability

Not applicable.
